# 

**Published:** 1890-09-20

**Authors:** 


### SATURDAY, SEPTEMBER 20th, 1890.

**The London is Bold** - 369
**Words of Consolation.**—Salvation - 370
**The Doctor's Pulpit.**—Cancer Curers - 371
**Working Women in Large Towns.**—X.—Domestic Servants (*continued*) - 372
**The History and Capabilities of Herbal Simples.**—XVIII.—Chervil—Celandine; the Greater - 373
**Sympathetic Surgery.**—I. - 374
**Everybody's Page** - 375
**Notes and News** - 376
**The Story of the Insane from Year to Year** - 377
**Annotations.**—Duelling and Development—What the Vegetarians Want to Know—Kent Christians - 378
**The Practitioner's Mirror and Retrospect:**—
Medicine — Chronic Bright's Disease, 379; Diabetes—Recent Research - 380
Surgery—Ear Disease, 381;—Nephrolithotomy - 382
**Leprosy Investigation** - 38[illegible]
**Passing Topics.**—A Question about Out-patients - 384
**General Charities** - 384

### EXTRA SUPPLEMENT—THE NURSING MIRROR.

**En Passant.**—Fresh Air—Women Inspectors—Nursing in America—Bath Hospital—Sister Rose Gertrude—Menial Work—Native Medical Women in India—Nursing as a Profession - ciii
**Sketches of Insanity.**—XI.—Epileptic Insanity (*continued*) civ
**Appointments** - civ
**Are Nurses Sweated?** - cv
**Nurses on their Travels** - cv
**Everybody's Opinion.**—Lady Nurses and Domestic Servants - cvi
**My Staff** - cvi
**Notes and Queries** - cvi
**Vacancies** - cvi
**Amusements and Relaxation** - cvi

